# Preparing for a trial to test a postpartum weight retention intervention among low income women: feasibility of a protocol in a community-based organization

**DOI:** 10.1186/s12905-018-0517-0

**Published:** 2018-01-25

**Authors:** Charmaine Wright, Marjie Mogul, Glamarys Acevedo, Jaya Aysola, Florence Momplaisir, Sandy Schwartz, Judy Shea

**Affiliations:** 1 Division of General Internal Medicine, Blockley Hall, 423 Guardian Dr, Philadelphia, PA 19104 USA; 2 Maternity Care Coalition, 2000 Hamilton Avenue, Suite 205, Philadelphia, PA 19130 USA; 3 Drexel School of Medicine, Department of Medicine, Infectious Diseases, Partnership Comprehensive Care Practice, 1427 Vine Street, 2nd Floor, Philadelphia, PA 19102 USA

## Abstract

**Background:**

Postpartum weight retention (PPWR) causes intergenerational harm, negatively affecting a mother’s cardiovascular health and ability to have future healthy pregnancies. Low-income minority women are at highest risk for PPWR with little guidance concerning timeline or strategy to lose weight after delivery. An academic-community partnership conducted observational and focus group work to develop an intervention for PPWR among low-income mothers. This study’s objective is to determine the feasibility of implementing a PPWR intervention trial in partnership with a community-based organization (CBO) serving low-income families with social service support.

**Methods:**

We analyzed five implementation outcomes in this feasibility study: acceptability, adoption, appropriateness, penetration, and sustainability. Other secondary outcomes were the change in psychosocial and clinical outcomes from baseline to one year following the intervention delivery.

**Results:**

An academic-community partnership developed and piloted a postpartum weight retention intervention among 17 participants that included 1) six weeks of interactive daily health texting, 2) exercise assistance with baby carrier, home exercise program, and pedometer provision, 3) two live healthy eating and baby feeding workshops, and 4) two 45-min home visits over one year to provide social support and acquire followup data. Implementation outcomes demonstrate an intervention supported by the organization and accepted by end-users, with increased capacity of the CBO to test and deliver an effective intervention. Weight loss was achieved by the majority of participants at one year (Md − 5 pounds (IQR = − 14.5 - 0.3).

**Conclusion:**

We made protocol enhancements to the developed intervention based on the analysis of this study, and now prepare for a funded randomized controlled trial (RCT) in a community-based setting. Our central hypothesis is that low-income women who participate in a multi-component, low cost-intervention delivered by a CBO will have less postpartum weight retention than those women who do not participate in the program.

**Trial registration:**

The trial was retrospectively registered, ID NCT02867631, 8/11/16.

## Background

Postpartum weight retention (PPWR) is associated with future unhealthy pregnancies, long-term obesity, and chronic cardiovascular disease [1–4]. Despite the growing awareness of the dangers of postpartum weight retention, neither an ideal time nor method to return to pre-pregnancy weight has been established in the literature [4–8]. Of additional concern is the recent demonstration of the postpartum period as a life stage characterized not only by weight retention, but also by susceptibility to excessive weight gain [9, 10]. Though Hispanic and African-American low-income mothers are at greatest risk for PPWR, postpartum gain, and adverse future birth outcomes, it remains a poorly-understood phenomenon. PPWR is thought to be the result of a variety of environmental, socioeconomic, and educational factors including starting pregnancy at unhealthy weight, gaining too much weight during pregnancy, maternal stress, and lack of breastfeeding and knowledge about healthy behaviors [11–14].

In the literature, several postpartum weight retention interventions have been studied. Intervention delivery was undertaken by a range of health professionals in various settings, but no definitive pattern was seen in successful interventions, and few took place in community-based settings [15–17]. Phelan et al. recruited low-income mothers from Women Infant and Children (WIC) Supplemental Nutrition Program offices and was the first to implement an internet-based treatment program with monthly group sessions [18, 19]. Van der Pligt et al. implemented a largely online intervention with dietician counseling by phone and calorie tracking by app among first time mothers in Australia, and also encouraged the use of less burdensome interventions that do not require face-to-face contact among busy new mothers [20]. Fernandez et al. implemented a trial testing an electronically-mediated behavioral intervention in an urban population of mothers recruited from an obstetric practice [21]. All these studies required the use of a smart phone or computer to participate.

In addition to focusing on socioeconomically disadvantaged mothers [22–24], racial and ethnically-sensitive approaches among Hispanic and African-American mothers have been piloted [20, 22, 23, 25–30]. Chasan-Taber et al. focused on Hispanic mothers in Massachusetts in an effort to test a culturally and linguistically modified intervention utilizing multimodal sources of counseling and goal setting for new mothers [22]. With efforts to prevent postpartum weight retention in African American mothers meeting multiple challenges, Herring et al. effectively recruited African American Medicaid recipients to a positive intervention study that included Facebook support and health coaching provided by telephone, with very little loss to followup at six months [28]. Though it seems clear that diet alone or a combination of diet and exercise will allow mothers to achieve postpartum weight loss as demonstrated by Cochrane Review [31], it remains unclear as to the optimal setting, delivery method, method of contact, or intervention length. As low-income mothers often have little health care beyond a recommended six week post-delivery appointment [24], this study will provide some clarity on how we might provide longer-term postpartum care for women in community based settings.

As part of a larger research project to reduce postpartum weight retention by determining the effectiveness of a community-based intervention among low income mothers, the University of Pennsylvania research team partnered with a community-based organization providing social services to low income women with a home visitation model implemented by community health workers (CHWs). This partnership performed observational [13] and focus group work (in press) leading to an holistic intervention informed by the tenets of community-engaged research (CER) and the theoretical framework of Ajzen’s Theory of Planned Behavior (TPB) [32, 33]. Specifically, we aimed to improve participants’ perceived behavior control (self-efficacy), subjective norms, and attitudes regarding postpartum weight management by developing four intervention components: 1) Motivational appeals to augment self-efficacy delivered by daily health texting for a period of six weeks; 2) Provision of environmental aids to improve self-efficacy for exercise and attitudes towards exercise by encouraging daily at-home physical activity with a twenty-minute baby carrier exercise program and continuous pedometer usage; 3) Task-oriented support to improve knowledge and attitudes for healthy eating and infant feeding through two live ninety-minute classes; and 4) Provision of peer support to combat perinatal depression, influence subjective norms, and enhance perception of social support with class and two forty- five-minute home visits over one year following delivery. The curriculum for the task-oriented support was delivered during the two classes and health texting. It directly answered the call for help we demonstrated with focus group work among staff and clients of the CBO. In regards to postpartum health, women asked for structure, social support, assurance they would not injure themselves or their babies, and time saving strategies to eat and exercise in a healthy manner after delivery.

The curricula therefore included meal planning, avoidance of sugar-sweetened beverages, and actionable healthy texts that include 100-cal snack ideas. The healthy feeding curricula delivered task-oriented instruction focused on lactation support provided in class and by phone if requested, bottling education, and goals of infant solid food introduction. The addition of the infant feeding instruction was prompted by focus group work demonstrating many client’s requests to know more about ways to combat pediatric obesity and its intergenerational transmission [14, 34].

The current study focuses on determining the feasibility of implementing a randomized clinical trial of these intervention components among clients of the partnering community-based organization, primarily focusing on five out of eight implementation outcomes defined by Proctor et al. including acceptability, adoption, appropriateness, penetration, and sustainability [35]. Since we were adjusting protocol in a dynamic fashion to maximize feasibility, we did not test fidelity or cost in this study.

## Methods

### Overview

The intervention components are informed by the Theory of Planned Behavior as described. The academic-community partnership developed the overall study design, and chose the outcomes based on a literature review of the behaviors we aimed to change: increase in meal planning, increase in eating fruits and vegetables, decrease in sugar-sweetened beverages, decrease in feelings of social isolation, increase in walking and incorporating the baby into exercise, and increase in breastfeeding time. The primary outcome is therefore postpartum weight retention (calculated as an absolute difference in pounds between one year postpartum and initial study weight obtained at 6–12 weeks postpartum). The secondary outcomes will be improved dietary composition (by dietary recall, from our validated nutrition knowledge and behavior psychosocial constructs [13]), decreased risk of depression (by Edinburgh screen [36]), improved breastfeeding time (in weeks) and maintained infant growth (weight for length).

### Participants

The CHWs were engaged at staff meetings and through email, and encouraged to refer their pregnant and postpartum clients to the study team for a feasibility study of the intervention. The target population was a convenience sample of low-income postpartum clients of the CBO, 100% of which are WIC-eligible. Inclusion criteria included English or Spanish-speaking recently postpartum non-pregnant participant, with self-reported pre-pregnancy BMI ≥ 18.5 kg/m^2^, delivery of single, live term (> 37 weeks gestation) infant, and cell phone with text messaging capabilities (92% clients in preliminary work). Women were excluded from the pilot study if their pre-pregnancy BMI was < 18.5 kg/m^2^ (underweight). Additionally, we terminated a mother from the study if BMI was not maintained above 18.5 kg/m^2^, or infant weight loss was noted at any of the weigh-ins, with referral to her healthcare provider. Our goal was to recruit 15 people which represented 10% of our initial proposed sample size needed for the RCT (*n* = 150), to detect a difference of two pounds of weight between groups with a standard deviation of four.

### Conduct of pilot intervention

Over three months, 26 women were referred of which 17 were eligible and in the window of 6–12 weeks postpartum at the time of the first study session. This window was chosen to engage women in the intervention after attending their recommended six week postpartum follow up visit with their health care provider to ensure safety to exercise. Women were recruited from 3 of the CBO sites and attended an initial ninety-minute class at their neighborhood site. They returned for a follow up class six weeks later, with 45-min home visits at six and twelve months post-delivery. Cab rides to attend classes were provided, as well as childcare for older children, and an invitation for a partner or support person to accompany the participant. The most intensive part of the intervention was between the two classes during which time women were encouraged to use the baby carrier with associated 20-min exercise program and pedometer while receiving and answering forty two days of text messages. The timeline for receipt of these intervention components is noted in Table 1.

**Table 1:** Intervention Components

Event	T0	T6 weeks	T6 months	T12 months
Class: Healthy Eating and Feeding	X	X		
Receipt of home exercise aids: pedometer, baby carrier, and home exercise program booklet	X			
Daily Motivational Texting and self-report of pedometer steps and time spent using baby carrier	X	X		
Home Visits			X	X
Vitals and Assessment	X	X	X	X

### Measures

1. Acceptability of the intervention trial was defined in a few ways: 1) rate of initial enrollment of those referred; 2) rate of attrition over the one year follow-up period; 3) engagement with the environmental aids given (time spent using the baby carrier, exercise program, and pedometers and rate of texting back study data); 4) post intervention self-reported utilization of environmental aids; and 5) post intervention reported satisfaction; and 6) positive attitudes towards planned randomization.
2. Adoption was defined qualitatively by the CBO leadership’s support of the study as demonstrated by the internal and external communication of the intervention and organizational development around the study.
3. Appropriateness was defined qualitatively by the academic-community partnership as the ability to recruit and retain participants at a rate comparable to other community-based programs, with the use of one research assistant, an interventionist, and the current geographic resources.
4. Good penetration was defined as the widespread referral of clients from the eight geographic sites of the CBO.
5. Sustainability was defined as the ability to acquire grant funding for a larger RCT and the ability of the CBO to assume all administrative and logistical components of the intervention with minimal outside assistance.
6. Nutrition knowledge and psychosocial factors were evaluated by a comprehensive tool previously validated by the academic-community partnership [13], and was measured at baseline, six weeks, six months, and twelve months.
7. Vital sign assessment including maternal and child height and weight was measured at baseline, six weeks, six months, and twelve months.

### Data collection

A CHW and research assistant collected paper surveys at each class, and at six and twelve months postpartum. A script was written for both the classes and home visits to ensure fidelity of the delivered intervention. Anthropometric training was provided to the research assistant using the National Health and Nutrition Examination Survey videos and protocol to ensure the collection of research-standard measurements, including maternal weight on an electronic scale measured to the nearest 0.1 kg (Seca Robusta 213), and height measured by a portable stadiometer (Seca 213). The infant weight was acquired by measuring both mother and baby on the scale. Infant length was acquired using the Measure Mat II Infantometer (Hopkins Medical). Participants reported their pre-pregnancy weight and gestational weight gain. All survey data were entered into REDCap (R6.16.4) for later analysis by the Stata statistical package (STATA 13, college Station TX).

### Data analysis

Univariate analysis was completed with chi-squared (categorical variables) and t-tests (continuous variables) to determine differences among those participants who were retained and those who dropped out. Mean and median postpartum weight change was calculated with interquartile ranges.

## Results

Acceptability and penetration: During the intervention pilot, we focused on operations and protocol enhancement by looking at the implementation outcomes described. Initially, we enrolled 17 women, with 5/17 (30%) attrition by six weeks, 6/17 (35%) at 6 months, and 8/17 (47%) attrition at one year. For this initial pilot recruitment, we relied on referrals from community health workers, with disparate rates of referral across the neighborhood sites. For example all women came from 3 sites where the CHWs had a previous interest in health and nutrition and had an increased level of success with recruitment. 5–8 women were referred by these advocates monthly, and demographics mirrored those of the population the CBO serves and those studied in focus group work. At the time, 3 other sites were being renovated and the CHW’s were busy managing the transition beyond their normal caseload and didn’t refer any clients from these sites. Yet another site had an ongoing transition of leadership and did not refer any clients during the study period. In terms of engagement by the mothers, during the intensive portion of the intervention, 8/12 (66%) reported use of the baby carrier more than half the days of the week; 9/12 (75%) reported pedometer use more than half the days of the week. Of those followed through six months, 8/11 (73%) continued to use the baby carrier and pedometer provided at least once a week, and 7/11 (64%) viewed the packet of exercise, eating, and baby feeding tips at least once a week. Engagement with texting back footsteps and baby carrier time was variable, with 9/11 (82%) of women texting at least 3 times a week. 100% said they were satisfied and would tell a friend about the program. Those who dropped out of the study by one year were similar to those retained in regards to starting body mass index (BMI), self-efficacy, perinatal depression risk, racial/ethnic background, and days spent returning texts. Those who dropped out did have more children living at home as an important difference (Table 2). When asked about randomization, 9/11 (82%) of participants said they would be willing to be randomized as long as they still received the baby carrier and handout information.

**Table 2:** Pilot Study Participant Characteristics

	Population 1 year post-delivery (*n* = 9)	Drop out population (*n* = 8)
Initial age	25.6 (4.6) years	25.4(4.1) years
Race/Ethnicity:		
Black	78%	62.5%
Hispanic	12.5%	25%
White	12.5%	12.5%
Initial Body Mass Index (BMI)	30.7 (4.0) kg/m^2^	31.9 (4.1) kg/m^2^
Initial waist circumference	38.7(3.8) inches	38.5 (4.0) inches
WIC recipient	89%	88%
Number children at home	1.2	3.2
Days of return texting	14	15

Adoption, Appropriateness, and Sustainability: Organizational support by the CBO was strong. The study was featured on the CBO’s website, and in the annual report. A story from one participant was featured in a CBO-wide email and blog. We were given conference room space and use of the fleet of CHW vehicles to acquire study tools and transportation to study sessions at no charge. We were successful in acquiring a pilot grant from the University of Pennsylvania Implementation Science Working Group, and a larger foundation grant from the Aetna Foundation to fund a CHW turned part -time research assistant who was based at the CBO but met with the PI at the University of Pennsylvania once weekly thus providing a critical link in the partnership, increasing CBO capacity. We employed a local lactation consultant and interventionist. During the follow up phase, we were able to secure additional funds in the form of further foundation grants, one of which emanated from the CBO itself which allowed us to plan for an RCT of the intervention using the same staffing.

Psychosocial and weight assessment: Mean self-efficacy for healthy behaviors improved over the course of the six week intensive intervention for the 12/17 who were retained at six weeks(from 6.9 (2.7) to 5.2(2.6), *p* = 0.04). However, self-efficacy returned to baseline by six and twelve months without significant change. Of those retained, more than half (7/12, 58%) lost weight over the course of the six week intensive intervention (Md − 1.0 pounds (IQR = − 4.1-1.8)), with 7/9 (78%) losing at one year post-delivery (Md − 5 pounds (IQR = − 14.5 - 0.3)). 25% of women were at risk for perinatal depression at the start of the program, and this point prevalence remained stable over time.

## Discussion

Based on the results of the pilot intervention, we made key protocol changes to effectively deliver the components during RCT testing. For example, during the pilot, we relied on CHW referrals to generate our study subjects. However, they had a large number of responsibilities they accomplish for the CBO home visitation program, and recruitment was subject to the environment at the neighborhood site. Recruitment for our study was limited by the amount of time the CHWs had, renovation or leadership changes at the neighborhood site, and the severity of an individual community health worker’s case load, as well as personal interest in health and nutrition. Since the level of penetration and attrition during the pilot was unacceptable to us and more than the 40% often encountered by community-based intervention studies, for our RCT intervention study, we will instead use a database of all postpartum clients managed by the CBO to recruit our participants, with CHWs providing input about safety information or mental health status regarding the participant. This change will allow us to contact every postpartum client as potential study subjects, and will allow the CHWs to focus on their primary responsibility of direct service provision. We can also ensure better penetration by contacting pregnant women from all geographic sites. We now know the importance of budgeting future funding to allow certain CHWs to be designated wellness champions with a reduced case load to allow for research study participation and program implementation, ensuring sustainability of the program once implemented. We will improve our emergency contact procedures for depressed participants as we found such a high prevalence during the pilot, and we will incentivize engagement with the intervention by providing an extra gift card to the woman who texts back the most in her cohort.

We also decided to increase the dose of the intensive intervention by lengthening class time to 120 min and home visit education to 60 min as we saw a significant initial change in self efficacy which returned to baseline at six and twelve month follow-up. The weight outcomes at six and twelve months post-delivery helped us to determine effect size and plan for our sample size in the RCT. To detect a 4 pound difference in weight change at one year post delivery using the standard deviations acquired (8), we would need a control population of 85 and an intervention population of 85 (an increase in sample size over our originally planned sample size). Attrition rates also aided in planning for our proposed recruitment sample. We used several previously described strategies for retention of our subjects in this community setting and anticipate the attrition seen in other community-based interventions (no more than 40%) [37]. We employ CBO staff as research study staff and thought critically about the type and timing of incentives to aid in recruitment. We piloted multiple procedures for participant follow-up during the pilot, including collecting emergency contact information at the outset, maintaining contact over the course of the entire year by text messages, and incentivizing updating contact information. The use of all three brought about the highest level of retention in our final cohort of women.

## Conclusion

This pilot study was a key step in planning for an RCT to study efficacy and cost-effectiveness of the holistic intervention, thus deepening the knowledge of best practices that exists for the combat of postpartum weight retention and its intergenerational downstream effects. At every point, community insight and collaboration has been considered. We were encouraged by this feasibility study that serves as a proof of concept and readies us for further testing with improved implementation outcomes and sample size to detect differences among control and intervention groups. Additionally, in this small group of women, we were able to assist women in decreasing their weight in the postpartum period while increasing their self-efficacy, thus improving their personal and familial health.

## Abbreviations

BMI: Body mass index
CBO: Community-based organization
CER: Community-engaged research
CHWs: Community health workers
PPWR: Postpartum weight retention
RCT: Randomized controlled trial
TPB: Theory of Planned Behavior
